# Intraoperative radiotherapy after resection of newly diagnosed brain metastases in adult patients - results of a prospective phase II trial (INTRAMET)

**DOI:** 10.1007/s11060-026-05649-6

**Published:** 2026-05-28

**Authors:** Stefanie Brehmer, Gustavo R. Sarria, Sara Würfel, Ardita Sulejmani, Frank Schneider, Sven Clausen, Yasser Abo-Madyan, Arne M. Ruder, Elena Sperk, Nima Etminan, Frank A. Giordano

**Affiliations:** 1 Department of Neurosurgery, University Medical Centre Mannheim, Medical Faculty Mannheim, Heidelberg University, Theodor-Kutzer-Ufer 1-3, 68167 Mannheim, Germany; 2 Department of Radiation Oncology, University Hospital Bonn, University of Bonn, Bonn, Germany; 3 Department of Radiation Oncology, University Medical Centre Mannheim, Medical Faculty Mannheim, Heidelberg University, Theodor-Kutzer-Ufer 1-3, 68167 Mannheim, Germany; 4 Mannheim Cancer Centre, Clinical Trial Unit, University Medical Centre Mannheim, Medical Faculty Mannheim, Heidelberg University, Mannheim, Germany; 5 DKFZ-Hector Cancer Institute, German Cancer Research Centre, Heidelberg, Germany; 6 Mannheim Institute for Intelligent Systems in Medicine (MIISM), Mannheim, Germany

**Keywords:** Brain metastases, Intraoperative radiotherapy (IORT), Brain metastasis cavity irradiation

## Abstract

**Purpose:**

Brain metastases occur in approximately 20% of cancer patients. Surgical resection followed by radiotherapy is a standard approach for symptomatic lesions. Intraoperative radiotherapy (IORT) enables immediate irradiation of the resection cavity and may facilitate treatment integration. We conducted a prospective phase-II-trial to evaluate the efficacy and safety of low-energy X-ray IORT following resection of brain metastases.

**Methods:**

INTRAMET was an open-label, single-arm study enrolling patients with suspected brain metastases between 2017 and 2022. Following resection and frozen-section confirmation of metastatic disease, IORT was delivered to the resection cavity using a mobile low-energy X-ray device. A dose of 30 Gy was prescribed to the applicator surface. Primary endpoint was local control. Secondary endpoints included distant brain control, overall survival, time to initiation of subsequent systemic therapy, and treatment-related adverse events.

**Results:**

Thirty-five patients were included. Median follow-up was 25.7 months. Median patient age was 64 years, and 68.6% of metastases originated from lung cancer. Local control was 94.3% (95% CI, 82.9–98.8%), while distant brain control was 57.1% (95% CI, 40.7–72.4%). Median overall survival was 43.6 months. Radionecrosis occurred in 20% of patients, predominantly low grade; no grade 4 or 5 IORT-related toxicities were observed. Leptomeningeal dissemination outside the irradiated field occurred in 8.6% of patients. Median time to initiation of subsequent systemic therapy was 45 days.

**Conclusion:**

IORT to the resection cavity was associated with favorable local control and an acceptable safety profile. It may represent a feasible alternative to postoperative stereotactic radiosurgery in selected patients.

**Supplementary Information:**

The online version contains supplementary material available at 10.1007/s11060-026-05649-6.

## Introduction

Brain metastases (BM) occur in approximately 20% of all patients with cancer over the course of their disease [1]. Although overall cancer-related mortality has declined, survival in patients with BM is still typically measured in months [2]. This prognosis is driven by several factors. First, the presence of BM reflects an advanced stage of systemic disease [3]. Second, patients presenting with symptomatic BM often have a poor clinical status, making it difficult to distinguish between general disease-related decline and potentially reversible, lesion-specific neurological effects [4]. Finally, the blood–brain barrier limits the penetration of many conventional anticancer agents, preventing them from reaching therapeutic concentrations within the brain [5].

Several studies have demonstrated that BM can infiltrate up to 1.2 mm into the surrounding, non-resected brain tissue [6, 7]. Consequently, omission of adjuvant irradiation is associated with local recurrence rates exceeding 60% [8]. Although postoperative stereotactic radiosurgery (SRS) to the resection cavity has become a widely used standard adjuvant treatment approach [6, 7] local recurrence rates after single-fraction postoperative SRS have historically been reported to reach up to approximately 30%, particularly in larger resection cavities where dose constraints may limit adequate target coverage [8, 9]. More recent series using fractionated stereotactic radiotherapy (FSRT) have demonstrated improved local control outcomes [10, 11]; however, these approaches differ conceptually and logistically from single-session strategies such as intraoperative radiotherapy (IORT), which delivers adjuvant treatment immediately at the time of resection.

Intraoperative delivery of adjuvant radiation may reduce the interval between neurosurgical resection and the initiation of subsequent systemic therapies. Immediate intracavitary irradiation can be achieved either through temporary or permanent implantation of radioactive isotopes (¹³¹Cs or ¹²⁵I) [9, 12–15] or by using intraoperative radiotherapy (IORT) [16–22]. The latter employs kilovoltage X-ray irradiation, characterized by rapid dose absorption and a high relative biological effectiveness [23]. Compared with SRS, kilovoltage irradiation allows high-dose delivery to the target volume while minimizing exposure of adjacent healthy brain tissue and organs at risk [24]. From a radiation safety perspective, shielding requirements are comparable to those of fluoroscopy [25], supporting the feasibility of IORT in the neurosurgical operating environment.

We investigated the efficacy and safety of adjuvant IORT in selected patients with BM and report the results of a prospective, single-arm phase II trial (INTRAMET).

## Methods

### Study design, ethics and patient selection

INTRAMET was a prospective, single-arm, open-label phase II study designed to evaluate the efficacy and safety of kilovoltage IORT as adjuvant treatment following resection of BM.

The study was planned to enroll 50 patients, based on anticipated outcomes comparable to those reported by Brown et al. and Mahajan et al. in two landmark trials of adjuvant stereotactic radiosurgery (SRS) following BM resection, which demonstrated 1-year local control rates of 60–72% [26, 27]. Patients with suspected BM and anticipated gross total resection on contrast-enhanced T1-weighted magnetic resonance imaging (MRI) were eligible. Inclusion criteria comprised age ≥ 18 years, KPS ≥ 50, histopathological confirmation of metastasis by intraoperative frozen section, and technical feasibility of IORT as determined intraoperatively. Exclusion criteria included contraindications to surgery or MRI, meningeal involvement, pregnancy or breastfeeding, psychiatric or social conditions impairing protocol compliance, or a predicted maximum IORT dose exceeding 8 Gy to critical organs at risk. Prior irradiation of the brain was not considered an exclusion criterion; however, the metastasis intended for IORT had to be treatment-naïve, which precluded prior whole-brain radiotherapy or stereotactic radiosurgery to the target lesion. Although prior cranial radiotherapy was permitted per protocol, no patient in this cohort had received any form of brain radiotherapy before IORT.

### Preoperative planning

Preoperative contrast-enhanced MRI scans (T1-weighted magnetization-prepared rapid acquisition, 1-mm slice thickness) were used for neuronavigation and treatment planning (Brainlab AG, Munich, Germany). Distances between the tumor margin and adjacent organs at risk were assessed preoperatively, and dose distributions of a centrally positioned spherical irradiation applicator within the anticipated resection cavity were considered.

### IORT procedure and workflow

Following tumor resection and achievement of hemostasis, the surgical cavity was assessed intraoperatively to determine its diameter. Distances from the cavity margins to the brainstem and optic pathways were measured and documented using intraoperative neuronavigation.

IORT was delivered using the INTRABEAM system (Carl Zeiss Meditec AG, Oberkochen, Germany), a mobile miniaturized X-ray device with a nominal output of 50 kV. Applicator size was selected according to cavity geometry to ensure optimal surface contact. A single layer of absorbable hemostatic material was permitted. A dose of 30 Gy was prescribed to the applicator surface (0 mm). Accounting for the increased relative biological effectiveness (RBE = 1.3), conservative dose constraints were applied, allowing a maximum single-fraction dose of 8 Gy to the brainstem and optic pathways.

### Follow-up

All patients underwent early postoperative MRI within 72 h. Gross total resection was defined as absence of residual contrast-enhancing tumor on this early postoperative scan. Follow-up visits were scheduled at 2 and 6 weeks after surgery and every 3 months thereafter, including neurological examinations and MRI with advanced sequences such as perfusion and diffusion imaging. Patients were followed until death or last follow-up. Causes of death were determined during systematic follow-up using clinical follow-up data, medical records, and available cancer registry information, with additional information obtained from treating physicians or relatives when required.

### Endpoints

The primary endpoint was local control rate (LCR), defined as the absence of in-cavity or marginal contrast-enhancing lesions suggestive of recurrence on serial contrast-enhanced T1-weighted MRI, assessed according to the Response Assessment in Neuro-Oncology Brain Metastases (RANO-BM) criteria [28] In cases of new cavity enhancement, short-interval follow-up MRI was performed. Dynamic perfusion imaging was obtained at each follow-up time point. To support differentiation between local recurrence and grade 1 radionecrosis, relative cerebral blood volume (rCBV) ratios were calculated between enhancing regions and contralateral normal white matter. In accordance with institutional practice and previously published perfusion MRI studies, lesions with rCBV ratios < 2 were considered suggestive of radionecrosis in the absence of progressive imaging findings on serial follow-up examinations [29–34]. In equivocal cases, short-interval follow-up MRI after approximately 6 weeks was performed. Censoring for LCR analysis occurred at whole-brain radiotherapy, death with documented follow-up, study completion, or last available MRI in patients lost to follow-up.

Secondary endpoints included distant brain control rate, defined as the absence of new parenchymal BM, overall survival (OS), BM–specific mortality, patient-reported quality of life assessed using European Organisation for Research and Treatment of Cancer questionnaires [35, 36], neurocognitive performance assessed with the German Neuro-Oncological Working Group NOA-07 battery [37], systemic disease mortality, and time to subsequent systemic therapy. Adverse events were graded according to the Common Terminology Criteria for Adverse Events version 5.0, with cumulative rates of early (< 3 weeks) and late (> 6 months) events reported. Neurocognitive and quality-of-life outcomes will be presented separately.

### Exploratory analysis of time to systemic therapy

To contextualize the observed time to initiation of subsequent systemic therapy, an exploratory retrospective analysis was conducted using an institutional cohort of patients who underwent resection of BM followed by conventional postoperative radiotherapy during a comparable time period. This analysis was not prespecified in the study protocol. Data were collected retrospectively, and no matching or adjustment for potential confounders was performed. Given the non-randomized design and differences in follow-up, this comparison was intended for descriptive purposes only and not for causal inference.

### Statistical analysis

Sample size calculation was based on A’Hern’s single-stage design [38]. Assuming a null hypothesis local control rate of < 60%, an alternative hypothesis of > 80%, a one-sided alpha of 5%, and 90% power, a sample size of 45 patients was required. Allowing for an anticipated 10% dropout rate, the target enrollment was set at 50 patients. These assumptions were based on outcomes reported in prior postoperative SRS trials and an earlier IORT study [16, 26, 27]. According to the pre-specified A’Hern single-stage design, recruitment could be discontinued once the lower bound of the observed 95% confidence interval exceeded the predefined alternative hypothesis threshold.

Statistical analyses were performed using SPSS version 28 (IBM Corp., Armonk, NY, USA). Group comparisons were conducted using t-tests, Fisher’s exact tests, or Fisher–Freeman–Halton tests as appropriate. Survival and control outcomes were analyzed using the Kaplan–Meier method and compared with the log-rank test.

## Results

### Patients and treatments

In total, 43 patients were screened, of whom 35 were enrolled in the study (CONSORT flow diagram shown in Online Resource 1, Figure S1). Enrollment was discontinued after the pre-specified statistical criterion for study success had been met according to the A’Hern single-stage design. Median follow-up was 25.7 months (range 0.8–64.5). Median patient age was 64 years (range 45–85), and median KPS was 80 (range 50–100), with 74.3% of patients having a KPS ≥ 80. Median tumor volume was 5.9 cm³ (range 1.2–43.1 cm³). Most BM originated from lung cancer (68.6%). Median graded prognostic assessment (GPA) score was 2.5 (range 0.5–3.5). Baseline patient and tumor characteristics are summarized in Table 1.

**Table 1:** Patient and tumor baseline features

		Median [Min-Max] or n (%)	
Gender		16 (45.7%)	
Female		19 (54.3%)	
Male		64 [45-85]	
Age			
ECOG	KPS		
0	100	15 (42.9%)	3 (8.6%)
	90		12 (34.3%)
1	80	18 (51.4%)	11 (31.4%)
	70		7 (20.0%)
2	60	2 (5.7%)	1 (2.9%)
	50		1 (2.9%)
GPA		2.5 [0.5-3.5]	
Total number of brain metastases at diagnosis			
1		27 (77.1%)	
2		5 (14.3%)	
3		3 (8.4%)	
Previously known primary		18 (51.4%)	
Patients with indication for systemic cancer treatment after brain metastasis surgery		24 (68.6%)	
Primary cancer site			
Breast		1 (2.9%)	
CUP		3 (8.6%)	
GI		3 (8.6%)	
Kidney		3 (8.6%)	
Lung		24 (68.6%)	
Ovary		1 (2.9%)	
Histology			
Adeno		20 (57.1%)	
Clear cell		3 (8.6%)	
ERneg, PRneg, HER2pos		1 (2.9%)	
Hepatoid Adeno		1 (2.9%)	
Serous Adeno		1 (2.9%)	
Squamous		5 (14.3%)	
Undifferentiated		4 (11.4%)	
Deep lesion		15 (42.9%)	
Eloquent lesion		15 (42.9%)	
Median metastasis volume (cm³)		5.9 [1.2-43.1]	
Median FLAIR volume (cm³)		110.3 [14.4-192.6]	
Applicator size (cm)			
1.5		16 (45.7%)	
2.0		13 (37.1%)	
2.5		4 (11.4%)	
3.0		2 (5.7%)	
Mean radiation time [mm:ss]		16:55 [08:24-40.57]	
Total resection		34 (97.1%)	
Seizures at diagnosis		13 (37.1%)	
Follow-up [Months]		25.7 [0.8-64.5]	
Patients alive		18 (51.4%)	

All patients treated with 30 Gy prescribed to the applicator surface. KPS/ECOG was assessed at baseline visit. Almost all patients were pretreated with dexamethasone

Abbreviations: ECOG, Eastern Cooperative Oncology Group; CUP, Cancer of unknown primary; GI, Gastrointestinal; ERneg, Estrogen receptor negative; PRneg, Progesterone receptor negative; HER2pos, human epidermal growth factor receptor 2 positive

The median applicator diameter was 2.0 cm (range 1.5–3.0), and the mean irradiation time was 16:55 min (range 08:24–40:57). A substantial proportion of patients received contemporary systemic therapies during the course of disease, including immunotherapy, tyrosine kinase inhibitors, chemotherapy, and multimodal combinations, often administered in sequential lines reflecting current multimodal systemic treatment strategies. In total, 13 patients received immunotherapy at some point during follow-up. Detailed patient and treatment-related characteristics are provided in Online Resource 1, Tables S1 and S2.

### Primary and secondary endpoints

**Fig. 1:**
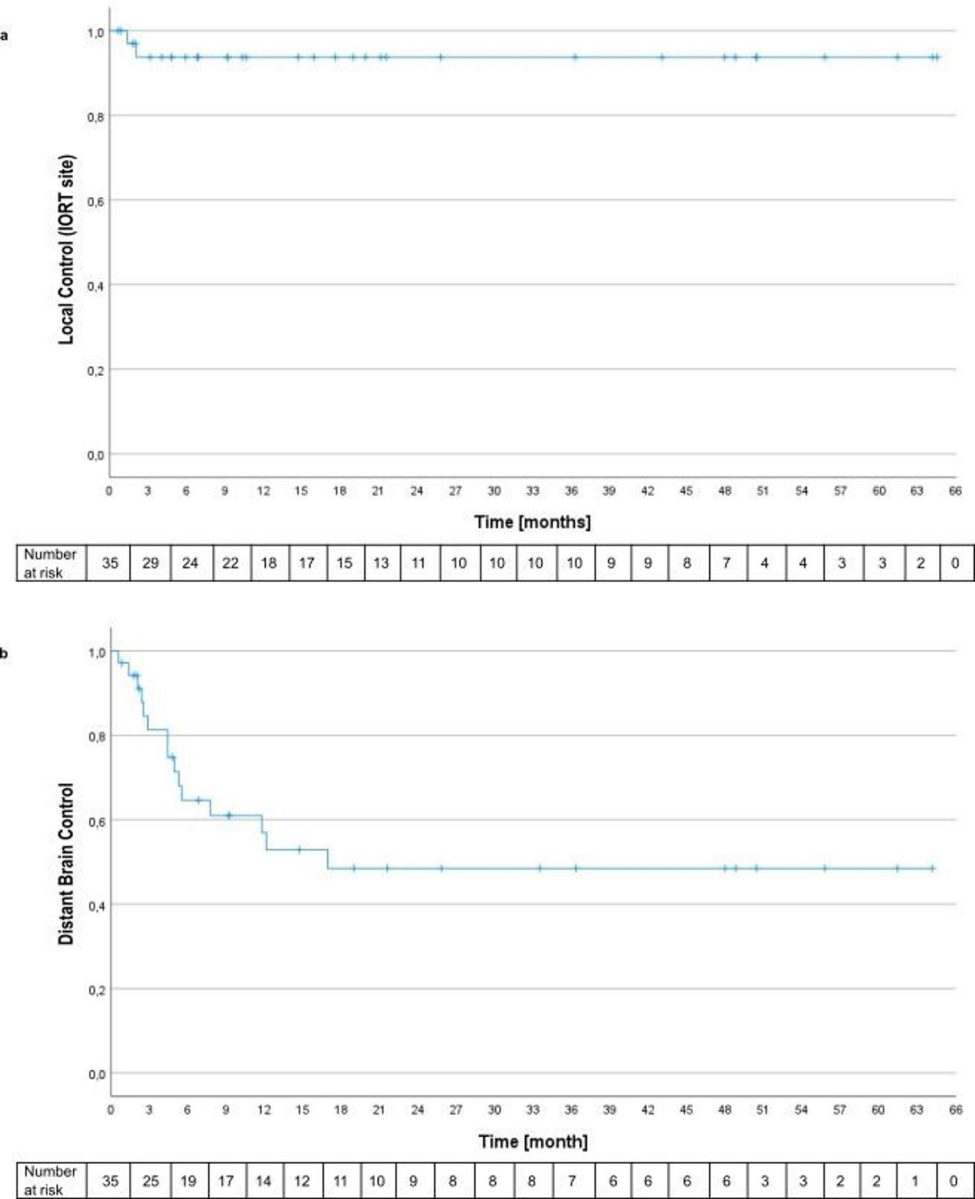
**A**. Local control of the surgically treated metastasis with IORT. Patients who received salvage whole-brain radiotherapy were censored at treatment initiation for local control assessment. **B.** Occurrence of new out-of-field metastases in the brain

LCR was 94.3% (95% CI 82.9–98.8%) at both 1 and 2 years, with only two local recurrences observed at 1 and 2 months after treatment (Fig. 1a). Patients who received salvage whole-brain radiotherapy (WBRT) during follow-up (*n* = 7; 20%), primarily for distant intracranial progression or leptomeningeal dissemination outside the IORT treatment field, were censored for local recurrence assessment at the time of WBRT initiation, as subsequent WBRT also irradiated the treated cavity. Patients with documented local recurrence prior to WBRT initiation were counted as local failure events and were therefore not censored.

The distant brain control rate was 57.1% (95% CI 40.7–72.4%), with new out-of-field BM occurring between 0.6 and 16.9 months after treatment (Fig. 1b). Three patients (8.6%) developed out-of-field leptomeningeal progression at 3, 38, and 44 months following surgery. The 1-year distant brain control rate was 62.9% (95% CI 46.3–77.3%), and the 2-year distant brain control rate was 57.1% (95% CI 40.7–72.4%).

### Patients were censored at their death or the end of follow-up for local and distant brain control assessment

**Fig. 2:**
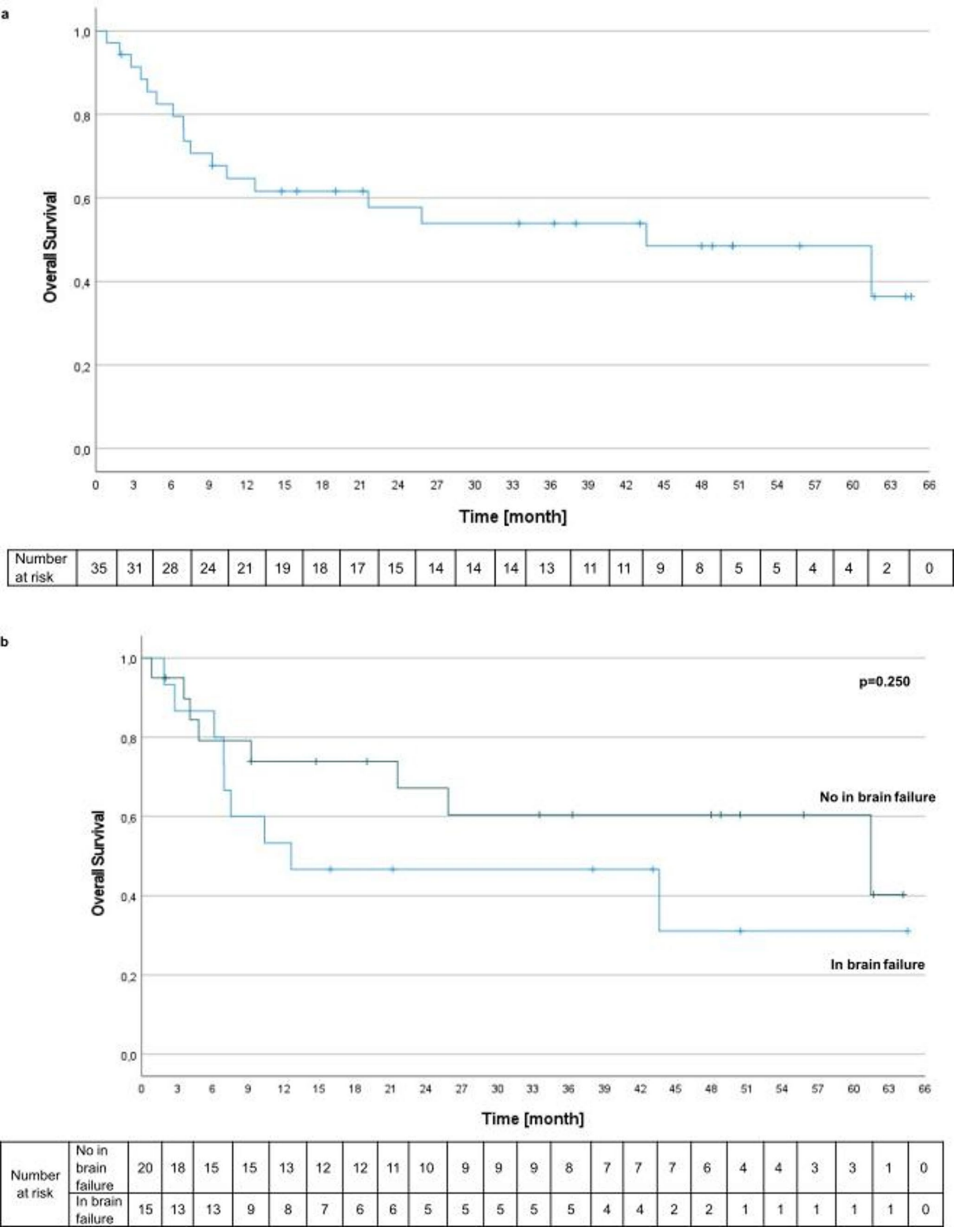
For survival assessment patients alive were censored at last follow-up **A**. Overall survival **B**. Overall survival categorized based on the emergence of new brain metastases during the course of disease

At the time of data analysis, 18 patients (51.4%) were alive. Of the 17 deceased patients, 2 (11.8%) died from cerebral progression, 4 (23.5%) from combined cerebral and systemic progression, 4 (23.5%) from systemic progression alone, and 7 (41.2%) from causes unrelated to the underlying malignancy (e.g., myocardial infarction, stroke, acute kidney failure, sepsis, or reactivation of a previous hematologic malignancy). Median overall survival (OS) was 43.6 months (95% CI 8.8–78.4; Fig. 2a). A non-significant trend toward improved survival was observed in patients without intracranial failure compared with those experiencing intracranial progression (*p* = 0.250; Fig. 2b).

Among patients requiring salvage WBRT, the median time from IORT to WBRT initiation was 147 days (range 20–601). Patients who did not require WBRT demonstrated a significantly longer median OS compared with those who did (42.4 months [95% CI 31.9–52.9] vs. 17.5 months [95% CI 3.8–31.1], *p* = 0.027). No significant survival differences were observed according to primary tumor histology (*p* = 0.618), receipt of immunotherapy (*p* = 0.928), presence of preoperative seizures (*p* = 0.169), or occurrence of radionecrosis (*p* = 0.214). Survival differences according to baseline KPS did not reach statistical significance (*p* = 0.056).

Time to initiation of a subsequent cancer therapy was 45.0 days [95% CI 35.1–54.8]. To provide context for this finding, an exploratory retrospective cohort of patients treated at our institution with resection followed by conventional external radiotherapy during a similar time period was analyzed. In this exploratory retrospective cohort, the median time to initiation of subsequent systemic therapy was numerically longer (56.6 days [95% CI 49.0–64.2]) compared with the IORT cohort (45.0 days [95% CI 35.1–54.8]). Given the retrospective and non-randomized nature of this comparison, including substantial differences in patient selection and baseline characteristics, these findings should be interpreted as descriptive and hypothesis-generating only. Cumulative rates of primary and secondary endpoints are summarized in Table 2. Individual patient outcomes are detailed in Online Resource 1, Table S3.

**Table 2:** Primary and secondary endpoint results

	*n* (%) or Median	95% CI
		Min-Max
Local progression rate	2 (5.7%)	1.2–17.1 [95% CI]
Distant brain progression rate	15 (42.9%)	27.6–59.3 [95% CI]
Leptomeningeal progression	3 (8.6%)	2.5–21.1 [95% CI]
Radionecrosis	7 (20%)	9.4–35.3 [95% CI]
New seizures	4 (11.4%)	4.0-24.9 [95% CI]
Time to further cancer therapy IORT [days]	45.0	35.1–54.8 [95% CI]
		16–120 [Min-Max]
Time to further cancer therapy retrospective cohort [days]	56.6	49.0-64.2 [95% CI]
		8-261 [Min-Max]
Time to RT retrospective cohort [days]	26.3	24.0-28.6 [95% CI]
		7-155 [Min-Max]

Control cohort: retrospective institutional cohort of surgically treated patients with brain metastases who did not undergo IORT during a similar treatment period

Abbreviations: IORT, intraoperative radiotherapy; RT, radiotherapy

### Safety

Safety outcomes were assessed according to CTCAE v5.0 criteria. No grade 4 or 5 IORT-related adverse events occurred during follow-up. A total of 12 grade 3 adverse events were observed, all of which were classified as at least possibly related according to CTCAE attribution criteria.

The overall incidence of radiation necrosis was 20%, comprising five grade 1, one grade 2, and one grade 3 events. Five patients were asymptomatic, while two were symptomatic. Symptomatic cases were managed with corticosteroids (*n* = 1) and bevacizumab (*n* = 1). One surgical resection was performed in an asymptomatic patient due to suspected tumor progression. No statistically significant associations between radionecrosis and evaluated clinical or treatment-related parameters (metastasis volume, applicator size, number of BM, resection status, immunotherapy or whole brain radiotherapy in further course of disease, BM location, BM histology) were identified. At last follow-up, no patient experienced persistent neurological impairment related to radionecrosis. Symptomatic cases showed clinical improvement under medical therapy, while asymptomatic cases remained clinically stable with either radiological stability or partial regression of imaging abnormalities without the need for specific treatment.

Postoperative seizures occurred in 28.6% of patients; on univariate analysis, eloquent tumor location was the only significant predictor (*p* = 0.008).

Table 3 summarizes all adverse events considered at least potentially related to IORT according to CTCAE v5.0 attribution criteria, while a comprehensive overview of all 231 recorded adverse events (89 grade 1, 80 grade 2, 52 grade 3, 6 grade 4, and 4 grade 5), including non–IORT-related events, is provided in Online Resource 1, Table S4.

**Table 3:** Potentially IORT-related adverse events by common terminology criteria for adverse events (V5.0) – occurrence <3weeks*, occurrence >6month~

		1	2	3	4	5	Relationship to IORT	Comments
Eye disorders	Diploic images	1~					unlikely	
Gastrointestinal disorders	Fecal incontinence	1					unlikely	
	Gastroesophageal reflux disease		1				unlikely	
General disorders	Fatigue	1~	1*				unlikely	
	Gait disturbance		2~				unlikely	
	Pain		1				unlikely	
Injury, poisoning and procedural complications	Dermatitis radiation	1					possible	
	Fall			1			unlikely	
Musculoskeletal and connective tissue disorders	Arthralgia		1				possible	
	Joint range of motion decreased	1					possible	
	Muscle weakness left-sided	1	2**				possible (1 × 1,2 × 2)	All IORT related weaknesses resolved completely within 3 months after surgery
	Muscle weakness right-sided		4***	1			possible (3 × 2); unlikely (2,3)	1 Patient suffered from Pembrolizumab induced adrenal insufficiency
Nervous system disorders	Ataxia	1	1				unlikely	
	Central nervous necrosis IORT site	5~	1	1			Definite (4 × 1), 2, 3; probable (1 × 1)	5 patients were asymptomatic, 1 patient received steroids, 1 bevacizumab, 1 patient received surgery due to suspected local progression
	Central nervous necrosis other site	1		1~			unlikely	
	Cerebrospinal fluid leakage	5***					possible	All resolved without intervention within 6 weeks after surgery
	Dizziness	1~					unrelated (1 × 1)	
	Dysesthesia	2**					possible	
	Dysgeusia	1*					Unlikely (1)	
	Dysphasia	2*	1*	1			possible (1 × 1,2); unlikely (1 × 1,3)	
	Headache	2~*	1				unlikely (1 × 2,1 × 1); possible (1 × 1)	
	Hydrocephalus			1			unlikely	
	Intracranial hemorrhage		1	1			unlikely (2,3)	
	Movements involuntary	1	1				unlikely	
	Paresthesia	1~					possible (2)	
	Peripheral sensory neuropathy		1~				unlikely	
	Seizure	10	5	5~~ ~*			possible (9 × 1,5 × 2,1 × 3); probable (1 × 3); unlikely (1 × 1,3 × 3)	13 patients presented initially with seizures at diagnosis, 4 patients had new seizures after surgery. The cumulative 20 events occurred in 13 different patients
Psychiatric disorders	Anxiety		1~				unlikely	
	Confusion	1	1~				unlikely	
	Insomnia		1				unlikely	
Renal and urinary tract disorders	Urinary incontinence		1*				unlikely	
Skin and subcutaneous tissue	Alopecia	2~	1~				possible (1 × 1); unlikely (1 × 1, 1 × 2)	
Vascular disorders	Thromboembolic Event		1				unlikely	

83 events were considered at least possibly related to IORT. No grade 4 and grade 5 event was in any form IORT related

Abbreviations: IORT, intraoperative radiotherapy

## Discussion

INTRAMET was a prospective phase II trial evaluating adjuvant IORT following resection of BM and demonstrates encouraging local control rates. Local control rates at both 1 and 2 years were 94.3%. Toxicity was low and comparable to that reported in postoperative SRS trials, with no IORT-related grade 4 or 5 adverse events observed during follow-up. A relatively short interval to initiation of subsequent systemic therapy was observed in this cohort.

The optimal adjuvant radiotherapy strategy following resection of BM remains controversial [39, 40]. While two pivotal randomized trials have established postoperative SRS as a standard of care, data evaluating preoperative SRS or IORT as alternative approaches remain limited, and direct comparisons between these strategies are scarce. In the trial by Brown et al., 91% of patients with tumors < 2.5 cm achieved local control at 12 months, while Mahajan et al. reported a local control rate of 86% for lesions < 3 cm [26, 27]. Compared with these well-established postoperative SRS approaches, outcomes in our study compared favorably in terms of local control, despite inclusion of lesions with tumor volumes up to 43 cm³. For adjuvant fractionated SRS, a large retrospective multicenter analysis including more than 550 patients reported a 2-year local control rate of 75% [11].

Preoperative SRS has recently gained attention as an alternative strategy. A prospective single-arm study including 48 patients reported a 6-month local control rate of 100% and a cumulative local control of approximately 91% over a median follow-up of 14.7 months. However, distant intracranial failure occurred in 63.6% of patients, leptomeningeal progression in 10%, and radionecrosis in 28.6% at 2 years. Importantly, this study applied volume-dependent dose constraints and had a relatively short follow-up, which does not reflect current survival expectations in this patient population [41]. A large multicenter cohort study comparing single-fraction versus fractionated preoperative SRS reported superior local control with fractionated schedules, likely attributable to higher cumulative doses (24 Gy in three fractions vs. 15 Gy single fraction), with 2-year recurrence rates of 2.9% and 16.3%, respectively. Radiation-related toxicity rates were low (7.4%) [42], underscoring that optimal dose concepts in the preoperative setting remain to be defined. The ongoing NRG BN012 trial is expected to provide further guidance.

From a logistical perspective, recent phase III data suggest that preoperative SRS does not necessarily delay surgery, alleviating a major concern associated with this approach [43]. Nevertheless, the need for preoperative imaging, treatment planning, and coordination may limit its widespread adoption, particularly in high-volume or resource-limited centers. In this context, IORT represents a practical alternative when available. Notably, unlike preoperative SRS, IORT allows histopathological confirmation prior to irradiation, which is particularly relevant in patients presenting with BM at first cancer diagnosis. Despite remaining limitations, a prospective comparison between preoperative SRS and IORT appears warranted as both modalities continue to evolve.

Brachytherapy offers another single-session adjuvant approach, with reported 1-year local control rates ranging from 82% to 100% in both retrospective and prospective series [9, 13–15, 44]. However, the use of radioactive isotopes entails substantial radiation safety requirements that are not universally available. Non-permanent implants necessitate subsequent removal, potentially requiring repeat surgery, and patient isolation during treatment may further limit feasibility and patient acceptance [12].

At present, the published clinical experience with IORT for BM has predominantly focused on the upfront perioperative setting, whereas data regarding salvage or re-irradiation applications remain limited.

The incidence of clinically relevant radionecrosis (≥ grade 2) in our study was 5.7%, consistent with rates reported in the literature [26]. Most cases were asymptomatic, radiographically detected grade 1 radionecrosis, which either resolved spontaneously or remained clinically inconsequential. Whether the applied dose distribution may also improve local control in larger BM remains uncertain and warrants further investigation.

Several factors may explain the favorable outcomes observed. Contemporary systemic therapies, including immunotherapy, targeted agents, and combination regimens, were frequently used in a heterogeneous and sequential manner in the present cohort and may have contributed to intracranial disease control and overall outcomes, although the study was not designed to formally assess their impact. Although the predominance of lung cancer histology could theoretically contribute to higher radiosensitivity, this explanation appears insufficient. Instead, dose escalation accounting for the higher relative biological effectiveness of kilovoltage irradiation, combined with the steep dose fall-off inherent to this modality, may represent an important contributing factor to the observed local control and toxicity profile [45–47]. Additionally, the low rate of leptomeningeal dissemination observed aligns with historical data [26] and suggests that irradiation of the entire surgical tract may be less critical than previously assumed [48, 49].

Patients with a KPS ≥ 50 were included, and no significant survival differences were observed across KPS strata. Importantly, KPS does not reliably distinguish between systemic disease burden and neurologic impairment attributable to the BM itself. In the latter scenario, substantial clinical improvement is often achieved following surgical resection and steroid therapy. Therefore, KPS alone should not preclude surgical intervention, and individualized decision-making remains essential.

In our cohort, the interval between surgery and initiation of subsequent systemic therapy appeared relatively short. In an exploratory retrospective institutional analysis conducted for contextual purposes, this interval appeared numerically shorter compared with patients undergoing conventional postoperative radiotherapy. However, this comparison was not prespecified, was not adjusted for potential confounders, and was based on a non-randomized retrospective cohort with substantial differences in patient selection, clinical characteristics, and follow-up. Accordingly, these findings should be interpreted as descriptive and hypothesis-generating only and do not allow for causal conclusions regarding treatment strategy. Nevertheless, the ability to deliver radiation at the time of surgery may represent a potential logistical advantage of IORT in selected clinical scenarios and warrants further investigation in controlled comparative studies.

IORT offers the potential for a single-session local therapy approach, particularly in patients with solitary BM. Completion of intracranial treatment at the time of surgery allows patients to rapidly resume daily activities or focus on systemic disease management. Avoidance of postoperative radiotherapy also reduces patient burden and may be especially beneficial for those with limited access to radiation oncology facilities. While preoperative radiotherapy may offer similar advantages, it is generally restricted to patients with histologically confirmed malignancies to ensure guideline-concordant treatment.

The mean irradiation time was 16:55 min, with most patients treated using 1.5- or 2.0-cm applicators. From a procedural perspective, IORT requires additional intraoperative steps, including device setup, applicator placement, and irradiation, which contribute to an extended intraoperative workflow. In our cohort, this did not result in relevant logistical constraints or procedure-related delays. This additional time was consistently manageable within the standard neurosurgical operating schedule. Emerging data suggesting comparable outcomes with lower prescription doses (e.g., 20 Gy) may substantially reduce beam-on and total operative time [50, 51].

At present, the availability of IORT for BM remains heterogeneous and is largely limited to specialized centers with access to dedicated intraoperative radiation equipment and interdisciplinary neurosurgical and radiation oncology workflows. As with other advanced radiotherapy techniques, implementation is likely influenced by institutional infrastructure, referral patterns, and local expertise. However, the comparatively limited shielding requirements of low-energy X-ray systems may facilitate integration into existing operative environments in appropriately equipped centers.

This study has several limitations. Its single-center, single-arm, open-label design limits generalizability and precludes direct comparisons with other adjuvant radiotherapy strategies. Although local control rates were high, the relatively small sample size limits the robustness and generalizability of the findings. In addition, recruitment was discontinued after fulfillment of the pre-specified statistical stopping criterion according to the A’Hern design. While this decision was prospectively defined within the study protocol, early termination of phase II trials may contribute to overestimation of treatment effects and limits the robustness of toxicity assessments. Furthermore, although neurocognitive function and quality-of-life data were prospectively collected, these longitudinal analyses were not included in the present report. Given the predominance of asymptomatic radionecrosis in this cohort, the clinical relevance of imaging-based treatment effects remains difficult to fully determine and warrants dedicated evaluation in future analyses. Accordingly, larger randomized trials with appropriate control groups are needed.

Dosimetric planning remains challenging due to limited intraoperative imaging quality and the absence of reliable Hounsfield units in cone-beam CT. Consequently, a pragmatic approach based on intraoperative distance measurements and water phantom calculations was employed, analogous to established brachytherapy workflows. Ongoing studies are exploring the role of advanced intraoperative imaging to further refine treatment planning [52].

Importantly, not all patients are suitable candidates for IORT. Brain shift, altered anatomy after decompression, or uncertainty in frozen-section diagnosis may preclude safe intraoperative irradiation, necessitating postoperative radiotherapy. Additionally, the predominance of lung cancer metastases and absence of melanoma BM in our cohort may limit applicability to patients with other primary tumor histologies.

In summary, while the results of INTRAMET are encouraging, they should be interpreted with caution. The favorable safety and efficacy profile of IORT has nonetheless provided the rationale for initiating a randomized phase III trial comparing IORT with standard postoperative radiotherapy, which is expected to open for recruitment shortly.

## Conclusion

Kilovoltage IORT represents a feasible adjuvant treatment option following resection of BM, achieving local control and toxicity outcomes comparable to those reported for postoperative stereotactic radiosurgery. In contrast to brachytherapy, it does not require handling of radioactive materials and allows completion of local intracranial treatment at the time of surgery. This single-session approach may be advantageous for selected patients, such as those with solitary lesions or in whom timely initiation of systemic therapy is a priority. A limitation of this study is the early termination of recruitment after the predefined statistical efficacy threshold had been met, resulting in a smaller cohort than originally planned. Further comparative studies are warranted to better define the role of IORT within the evolving multimodal management of BM.

## Supplementary Information

Below is the link to the electronic supplementary material.

Supplementary Material 1

## Data Availability

Research data supporting the findings of this study are published within the paper and in parts anonymized in the Online Resource. Additional research data are stored in an institutional repository and will be shared upon request to the corresponding author.
