# Supplementary material for: Intraoperative radiotherapy after resection of newly diagnosed brain metastases in adult patients - results of a prospective phase II trial (INTRAMET)
Source: J Neurooncol. 2026 May 28;178(1):28. doi: 10.1007/s11060-026-05649-6 (PMC13219085; doi:10.1007/s11060-026-05649-6)
Supplement: Supplementary file 1 — Supplementary Material 1 [file 11060_2026_5649_MOESM1_ESM.pdf]

Intraoperative radiotherapy after resection of newly diagnosed brain metastases in adult patients - results of a prospective phase II trial (INTRAMET)

Journal of Neurooncology

Stefanie Brehmer, MD[1*] Gustavo R. Sarria, MD[2]; Sara Würfel, MD[1]; Ardita Sulejmani, MD[1]; Frank Schneider, PhD[3]; Sven Clausen, PhD[3]; Yasser Abo-Madyan, MD[3]; Arne M. Ruder, MD[3]; Elena Sperk, MD[4]; Nima Etminan, MD[1]; Frank A. Giordano, MD[3,5,6*]

[1]Department of Neurosurgery, University Medical Centre Mannheim, Medical Faculty Mannheim, Heidelberg University, Mannheim, Germany

[2]Department of Radiation Oncology, University Hospital Bonn, University of Bonn, Bonn, Germany

[3]Department of Radiation Oncology, University Medical Centre Mannheim, Medical Faculty Mannheim, Heidelberg University, Mannheim, Germany

[4]Mannheim Cancer Centre, Clinical Trial Unit, University Medical Centre Mannheim, Medical Faculty Mannheim, Heidelberg University, Germany

[5]DKFZ-Hector Cancer Institute, German Cancer Research Centre, Germany

[6]Mannheim Institute for Intelligent Systems in Medicine (MIISM), Mannheim, Germany

***Corresponding author:**

Stefanie Brehmer, MD: Department of Neurosurgery, University Medical Center Mannheim, Theodor-Kutzer-Ufer 1-3 63167, Mannheim, Germany; Phone: +49 621 383 5968, Fax.: +49 621 383 2004; E-mail: stefanie.brehmer@umm.de; ORCID 0000-0001-9113-9865

Frank Giordano, MD; Department of Radiation Oncology, University Medical Center Mannheim, Theodor-Kutzer-Ufer 1-3 63167, Mannheim, Germany; Phone: +49 621 383 1549, Fax.: +49 621 383 2476; E-mail: frank.giordano@umm.de; ORCID 0000-0001-5243-4781

Supplemental Figure S1. Consort flow chart

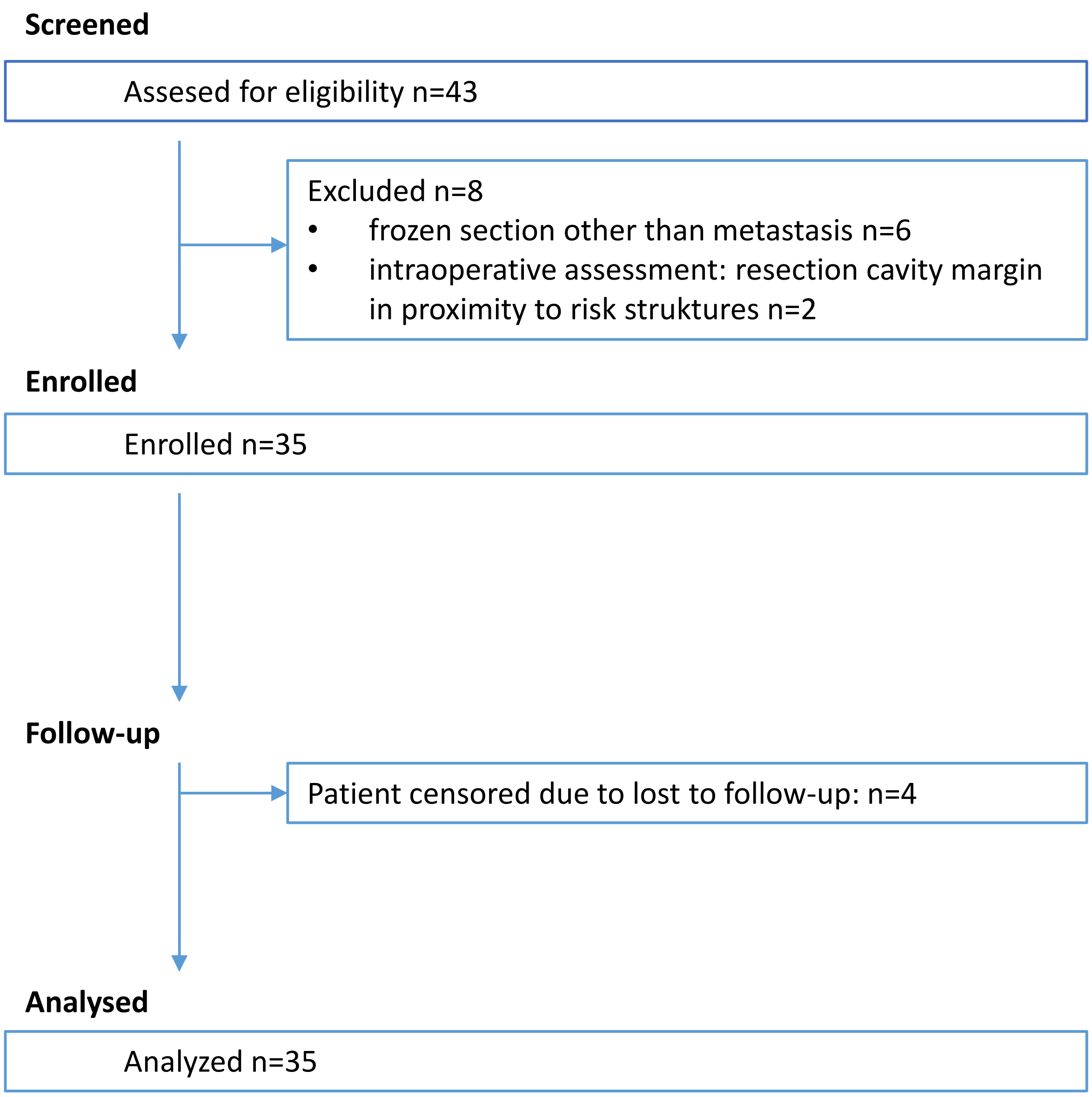

Supplemental Table S1 - patient baseline characteristics

Sex	Age	Karnofsky	ECOG	Number of brain metastasis at diagnosis	Primary cancer	GPA Index	primary previously known	Histology of the metastasis	Follow-up [month]	Target metastasis volume [cm³]	Target metastasis FLAIR volume [cm³]	Localization deep	Localization eloquent	Seizures at diagnosis	Indication for target metastasis resection
m	59	80	1	1	Lung	2,5	n	Squamous	5,09589041	43,1	110,3	y	n	n	Symptomatic space occupying metastasis (midline shift) with oncological necessity for histology
m	65	90	0	1	Lung	3	n	Adeno	61,6438356	15	179,2	n	n	n	Symptomatic space occupying metastasis (midline shift) with oncological necessity for histology
f	53	90	0	2	Lung	3	y	Adeno	43,5945206	1,79	54,8	y	y	n	Symptomatic space occupying metastasis with oncological necessity for histology
f	61	90	0	3	Lung	2,5	n	Adeno	64,5369863	1,22	60,4	n	n	y	Symptomatic space occupying metastasis with oncological necessity for histology
m	66	80	1	2	Lung	2	n	Adeno	61,4136986	18	136,3	n	n	n	Symptomatic space occupying metastasis (midline shift)
f	64	70	1	1	Lung	1,5	y	Adeno	64,1424658	3,74	51,8	n	y	y	Symptomatic space occupying metastasis
m	71	90	0	1	Lung	2,5	y	Squamous	21,5671233	4,22	96,2	n	n	y	Symptomatic space occupying metastasis with oncological necessity for histology
m	50	80	1	1	Lung	1,5	n	Hepatoid Adeno	6,90410959	17,7	177,8	y	n	n	Symptomatic space occupying metastasis (midline shift)
f	67	100	0	1	Lung	3	y	Squamous	55,7589041	5,88	14,4	n	n	n	Oncological necessity for histology
m	66	90	0	1	Lung	2,5	n	Adeno	50,4	1,99	135,8	n	y	n	Symptomatic space occupying metastasis with oncological necessity for histology
f	68	80	1	3	Lung	2,5	n	Undifferentiated	1,8739726	9,49	35,1	n	y	y	Symptomatic metastasis with neurological deficits
f	62	70	1	3	Ovary	1	y	Serous Adeno	12,230137	22,8	151,5	y	y	y	Symptomatic space occupying metastasis (midline shift)
m	66	90	0	1	Lung	2,5	n	Adeno	50,4328767	4,08	125,9	y	y	y	Symptomatic space occupying metastasis with oncological necessity for histology
f	68	80	1	1	CUP	2,5	n	Undifferentiated	48,7890411	21,4	86,1	y	n	y	Symptomatic space occupying metastasis with oncological necessity for histology
m	59	90	0	1	Lung	3,5	n	Adeno	47,9671233	7,47	68,7	y	y	n	Symptomatic metastasis with neurological deficits
f	80	70	1	1	Kidney	2	y	Clear cell	9,20547945	10,6	135,2	y	n	n	Symptomatic space occupying metastasis (midline shift)
m	46	60	2	2	Kidney	0,5	y	Clear cell	43,0684932	3,51	36,1	n	y	y	Symptomatic metastasis with neurological deficits
f	70	70	1	1	Lung	2,5	y	Squamous	2,20273973	5,08	31,9	y	y	y	Symptomatic space occupying metastasis with oncological necessity for histology
f	60	80	1	1	Kidney	2,5	y	Clear cell	33,5013699	11,3	122,2	y	n	n	Symptomatic space occupying metastasis (midline shift) with oncological necessity for histology
f	63	100	0	1	Lung	3	y	Adeno	38,0054795	2,2	27,6	n	y	y	Symptomatic metastasis with neurological deficits, oncological necessity for histology
m	67	50	2	1	GI	1	y	Adeno	1,77534247	12,7	159	n	n	n	Symptomatic space occupying metastasis (midline shift)
f	58	80	1	1	CUP	3	n	Undifferentiated	36,2958904	4,39	71,9	n	n	n	Symptomatic space occupying metastasis (midline shift) with oncological necessity for histology
f	73	70	1	2	Lung	2	y	Adeno	6,11506849	2,34	73,1	y	n	n	Symptomatic space occupying metastasis (midline shift) with oncological necessity for histology
m	53	80	1	1	Lung	2	n	Adeno	4,8	28,7	192,6	y	n	n	Symptomatic space occupying metastasis (midline shift) with oncological necessity for histology
m	85	90	0	1	Lung	2,5	y	Squamous	9,20547945	5,38	113,4	n	n	n	Symptomatic metastasis with neurological deficits, oncological necessity for histology
m	75	80	1	2	Lung	2	n	Undifferentiated	2,76164384	16,5	122,9	n	n	n	Symptomatic metastasis with neurological deficits, oncological necessity for histology
m	56	70	1	1	Lung	1	y	Adeno	25,8082192	4,1	179,6	n	n	n	Symptomatic space occupying metastasis (midline shift) with oncological necessity for histology
m	79	70	1	1	Lung	0,5	n	Adeno	2,00547945	16,4	82,3	n	y	y	Symptomatic space occupying metastasis (midline shift) with oncological necessity for histology
f	45	90	0	1	GI	3,5	y	Adeno	21,139726	5,11	32,1	n	y	y	Symptomatic metastasis with neurological deficits
m	70	80	1	1	GI	2	y	Adeno	18,969863	13,5	165	n	y	y	Symptomatic space occupying metastasis (midline shift) with oncological necessity for histology
m	66	90	0	1	CUP	2	n	Adeno	15,9123288	25,4	170,1	y	n	n	Symptomatic space occupying metastasis (midline shift) with oncological necessity for histology
m	74	90	0	1	Lung	2	n	Adeno	14,6958904	2,07	68,4	y	y	n	Symptomatic metastasis with neurological deficits
m	73	100	0	1	Lung	2	y	Adeno	6,9369863	3,3	37,6	n	y	n	Symptomatic metastasis with neurological deficits
f	65	90	0	1	Lung	3,5	n	Adeno	0,85479452	4,62	130,2	y	n	n	Symptomatic space occupying metastasis (midline shift) with oncological necessity for histology
f	46	80	1	1	Breast	3,5	y	ERneg, PRneg, HER2pos	9,20547945	18,2	141,8	n	n	n	Symptomatic space occupying metastasis (midline shift) with oncological necessity for histology

Abbreviations: m: male; f: female; CUP: cancer of unknown primary; GI: gastrointestinal; GPA: graded prognostic assessment; y: yes; n: no; ERneg: Estrogen receptor negative; PRneg: Progesterone receptor negative; HER2pos: human epidermal growth factor receptor 2 positive; FLAIR: fluid attenuated inversion recovery

Supplemental table S2 - patient treatment information						
Applicator size [cm]	Radiation Time [hh:mm:ss]	Complete resection	Begin further cancer therapy [days]	Type of further cancer therapy in follow-up	Immuno-therapy anytime in follow-up	Whole brain radiation therapy in follow-up [month]
2	00:20:31	n	36	Immunotherapy	y	2,1
2	00:20:23	y	84	Surgery of the primary	n	
2	00:20:31	y			n	19,8
2	00:20:31	y	80	Radiothery of the primary	y	
2,5	00:30:29	y	38	Immunotherapy	y	
2	00:17:01	y	28	Surgery of extracranial metastasis	n	
2	00:15:41	y	28	Immunotherapy	y	
2	00:15:51	y	41	Immuno-chemotherapy	y	
1,5	00:10:50	y	44	Surgery of extracranial metastasis	n	
1,5	00:10:49	y	58	Chemotherapy	n	
1,5	00:12:04	y			n	0,7
2	00:17:49	y	30	Chemotherapy	n	10,7
1,5	00:12:04	y	33	Surgery of the primary	y	
3	00:40:57	y			n	
1,5	00:12:13	y	47	Surgery of the primary	n	
1,5	00:11:01	y	23	Tyrosin-kinase-inhibition	y	
1,5	00:11:02	y	22	Immun-checkpoint-inhibition	y	
1,5	00:10:49	y			n	
3	00:40:00	y	38	Tyrosin-kinase-inhibition	y	
1,5	00:11:59	y			n	4,8
1,5	00:11:58	y			n	
1,5	00:08:24	y			n	
1,5	00:08:30	y			n	3,2
2	00:14:21	y	34	Immunotherapy	y	
2,5	00:26:00	y	104	Immunotherapy	y	
2	00:17:01	y	37	Surgery of the primary	n	
2	00:17:04	y			n	
2	00:17:07	y			n	
1,5	00:10:43	y	34	Chemotherapy + Bevazizumab	n	
2	00:12:56	y	49	Chemotherapy	n	
2,5	00:26:17	y	120	Surgery of extracranial metastasis	n	
1,5	00:10:55	y	19	Immuno-chemotherapy	y	
1,5	00:10:57	y	16	Chemotherapy	n	5,9
1,5	00:10:59	y			n	
2,5	00:26:08	y	49	Trastuzumab-Deruxtecan	y	
Abbreviation: y, yes; n, no						

Supplemental table S3 - patient outcome information

Seizures after surgery	Local progression (resection cavity) [month]	Regional progression (brain) [month]	Lepto-meningeal progression	Occurance of radiation necrosis [month]	Overall survival [month]	Vital status at end of study	Cause of death
n	1,4	1,4	n		7,5	Deceased	Cerebral progression
n			n			Alive	
y		16,9	y		43,6	Deceased	Stroke
n		2,4	n			Alive	
n			n	5,5	61,4	Deceased	Kidney failure
y			n			Alive	
n			n		21,6	Deceased	Systemic progression
n		5,5	n		6,9	Deceased	Systemic progression
n			n			Alive	
y			n			Alive	
n		0,6	n		1,9	Deceased	Systemic and cerebral progression
n		2,5	n		12,6	Deceased	Systemic and cerebral progression
y		12,1	n			Alive	
y			n			Alive	
n			n			Alive	
n		7,8	n		10,4	Deceased	Cardiac infarction
y		4,9	n			Alive	
n			n		4,1	Deceased	unknown
y			n	7,6		Alive	
y		4,4	y			Alive	
n			n		3,6	Deceased	Systemic progression
n			n	21,4 histologically proven		Alive	
n		2,9	n		6,1	Deceased	Cerebral progression
n		4,8	n		4,8	Deceased	Systemic progression
n		9,2	n		9,2	Deceased	Activation of preknown CLL
n	2,1	2,1	y		2,8	Deceased	Systemic and cerebral progression
n			n	9	25,8	Deceased	Activation of preknown T-cell-lymphoma
n			n			lost to follow-up	
n		11,8	n			Alive	
y			n	18,8		Alive	
n		4,4	n	2,8		Alive	
y			n	12,8		Alive	
n		5,3	n		6,9	Deceased	Systemic and cerebral progression
n			n		0,9	Deceased	Sepsis
n			n			Alive	

The patient lost-to follow-up was censored for outcome analysis
Abbreviations y: yes; n: no; CLL: chronic lymphtic leukemia

Supplemental table S4: Complete Adverse Events by common terminology criteria for adverse events grade (V5.0) including unrelated events<br>Occurance <3weeks*, occurance >6month~								
		**1**	**2**	**3**	**4**	**5**	**Relationship to IORT**	**Comments**
Blood and lymphatic system disorders	Anemia		1	2			unrelated	
Cardiac disorders	Atrial fibrillation	1					unrelated	
	Atrial flutter		1				unrelated	
	Left ventricular systolic dysfunction			1			unrelated	
	Right ventricular dysfuntion			1			unrelated	
	Myocardial infarction			3			unrelated	
	Palpitations	1					unrelated	
Ear and labyrinth disorder	Hearing impaired	1					unrelated	
Endocrine disorders	Adrenal insufficiency			2			unrelated	
	Hypopituitarism			1			unrelated	
	Hypothyroidism		1				unrelated	
Eye disorders	Blurred vision	1					unrelated	
	Diploic images	1~					unlikely	
	Dry eye	1					unrelated	
	Impaires visual field	1~					unrelated	
Gastrointestinal disorders	Anal fissure	1					unrelated	

	Constipation	1	1				unrelated	
	Diarrhea	3~	3~				unrelated	All patients received immunotherapy
	Dry mouth	1~					unrelated	
	Fecal incontinence	1	1*				unlikely	
	Gastroesophageal reflux disease		2				unlikely (1); unrelated (1)	
	Hemorrhoids	1					unrelated	
	Ileus				1		unrelated	Ileus due to per patient wish untreated colorectal cancer
	Mucositis oral	1					unrelated	
	Nausea	1	1				unrealted	
	Oral hemorrhage	1					unrelated	
	Stomach pain	1					unrelated	
General disorders	Fatigue	2~	3*	1~			unlikely (2x1,1x2); unrelated (2x2,1x3)	
	Gait disturbance		2~				unlikely	
	Infusion site extravasation		1				unrelated	
	Pain		1				unlikely	
	Vomitting		1				unrelated	
Hepatobiliary disorders	Cholecystitis			1			unrelated	
Immune system disorders	Autoimmune disorder				1		unrelated	Patient treated with immunotherapy
Infections and infestations	Bronchial infection			2~			unrelated	

	Enterocolitis infectious			1			unrelated	
	Esophageal infection			1			unrelated	
	Joint infection			1			unrelated	
	Lung infection		5	3		1	unrelated	Patient treated with immunochemotherapy
	Lymph gland infection			1			unrelated	
	Otitis media		1~				unrelated	
	Rash pustular		1~				unrelated	
	Tooth infection		1				unrelated	Patient treated with immunotherapy
	Thrush		1				unrelated	
	Unknown origin			2		1	unrelated	Patient treated with immunochemotherapy
	Skin infection			1			unrelated	
	Soft tissue infection			1			unrelated	
	Tracheitis			1~			unrelated	
	Urinary tract infection		1	2			unrelated	
Injury, poisoning and procedural complications	Dermatitis radiation	1					possible	
	Fall			1			unlikely	
	Fracture		1				unrelated	
Investigations	Cardiac troponin I increased	1					unrelated	
	Platelet count decreased		2				unrelated	

	White blood cell decreased			1			unrelated	
Metabolism and nutrition disorders	Dehydration			1			unrelated	
	Hyperuricemia			1			unrelated	
	Hypokalemia	1					unrelated	
	Hyponatremia				1		unrelated	Patient suffering from Pembrolizumab induced adrenal insufficiency
Musculoskeletal and connective tissue disorders	Arthralgie		3				possible (1x2); unrelated (2x2)	
	Back pain	2~					unrelated	
	Chest wall pain		2				unrelated	
	Joint range of motion decreased	1					possible	
	Muscle cramp	1					unrelated	
	Muscle weakness lower limp	2					unrelated	
	Muscle weakness left-sided	2	2**				possible (1x1,2x2); unrelated (1x1)	
	Muscle weakness right-sided	1~	4***	1			possible (3x2) unlikely (2,3); unrelated (1)	All IORT related weaknesses resolved completely within 3 months after surgery
	Pain in extremity	3					unrelated	
Neoplasm benign, malignant and unspecified	Activation preexisting					2	unrelated	1 patient had a previously diagnosed CLL (IORT treated primary: NSCLC), 1 suffered after renal

	lymphatic malignancy							transplantation from a post-transplant lymphoma (IORT treated primary: NSCLC)
	Intestinal polyp	1					unrelated	
Nervous system disorders	Ataxia	1	1				unlikely	
	Central nervous necrosis IORT site	5~	1	1			Definite; propable (1x1)	5 patients were asymptomatic, 1 patient received steroids, 1 bevacizumab, 1 patient received resection due to suspected local progression
	Central nervous necrosis other site	1		1~			unlikely	
	Cerebrospinal fluid leakage	5***					possible	All resolved without intervention within 6 weeks after surgery
	Dizziness	2~					unrelated (1x1); unlikely (1x1)	
	Dysesthesia	2**					possible	
	Dysgeusia	2*					Unlikely (1); unrelated (1)	
	Dysphasia	2*	1*	1			possible (1x1,2); unlikely (1x1,3)	
	Headache	3~*	2				unlikely (1x2,1x1); possible (1x1);unrelated (1x1,1x2)	
	Hydrocephalus			1			unlikely	

	Intracranial hemorrhage	1	1	1			unlikely (2,3); unrelated (1)	
	Movements involuntary	1	1				unlikely	
	Paresthesia	3~					possible (2); unrelated (1)	
	peripheral motor neuropathy	1					unrelated	
	Peripheral sensory neuropathy		3~				unlikely (1x2); unrelated (2x2)	
	Presyncope		1				unrelated	
	Seizure	10	5	5~~ ~*			possible (9x1,5x2,1x3); propable (1x3); unlikely (1x1,3x3)	13 patients presented initially with seizures at diagnosis, 4 patients had new seizures after surgery. The cumulative 20 events occurred in 13 different patients
	Stroke	1					unrelated	
Psychiatric disorders	Anxiety		2~				unlikely (1x2)	
	Confusion	1	1~				unlikely	
	Depression		1				unrelated	
	Insomnia		1	2			unlikely (1x2); unrelated (2x3)	
	Psychosis			1			unrelated	
Renal and urinary tract disorders	Acute kidney injury			1			unrelated	
	Urinary incontinence		1*				unlikely	

Respiratory, thoracic and mediastinal disorders	Bronchospasm			1			unrelated	
	Cough		1				unrelated	
	Dyspnea	1	2	3			unrelated	
	Pleural effusion		1				unrelated	
	Pneumonitis		2				unrelated	
	Pulmonary edema				2		unrelated	
Skin and subcutaneous tissue	Alopecia	3~	4~				unrelated (1,3x2)	
	Bullous dermatitis			1			unrelated	
	Eczema	3**	1~				unrelated	
	Pruritus	2~					unrelated	
	Rash maculo-papular		1				unrelated	
	Skin hyper-pigmentation	1					unrelated	
Vascular disorders	Hypertension		2~				unrelated	
	Lymphedema	1					unrelated	
	Thromb-embolic Event		1				unlikely	
	Vasculitis				1		unrelated	

The total number of adverse events was 230 (88 grade 1, 80 grade 2, 52 grade 3, 6 grade 4 and 4 grade 5) 85 events were not *unrelated* to IORT. No grade 4 and grade 5 event was in any form IORT related.
Abbreviations: IORT: intraoperative radiotherapy, CLL: chronic lymphatic leukemia, NSCLC: non-small-cell lung cancer
